# Transcriptomic analysis of α-synuclein knockdown after T3 spinal cord injury in rats

**DOI:** 10.1186/s12864-019-6244-6

**Published:** 2019-11-14

**Authors:** Hong Zeng, Bao-fu Yu, Nan Liu, Yan-yan Yang, Hua-yi Xing, Xiao-xie Liu, Mou-wang Zhou

**Affiliations:** 1 Department of Rehabilitation Medicine, Peking University Third Hospital, 49 North Garden Road, Beijing, 100191 China; 2 Department of Hand Surgery, Huashan Hospital, Fudan University, Shanghai, 200040 China

**Keywords:** Spinal cord injury, α-Synuclein, RNA-seq, Cholinergic synapse pathway, Neurogenesis

## Abstract

**Background:**

Endogenous α-synuclein (α-Syn) is involved in many pathophysiological processes in the secondary injury stage after acute spinal cord injury (SCI), and the mechanism governing these functions has not been thoroughly elucidated to date. This research aims to characterize the effect of α-Syn knockdown on transcriptional levels after SCI and to determine the mechanisms underlying α-Syn activity based on RNA-seq.

**Result:**

The establishment of a rat model of lentiviral vector-mediated knockdown of α-Syn in Sprague-Dawley rats with T3 spinal cord contusion (LV_SCI group). The results of the RNA-seq analysis showed that there were 337 differentially expressed genes (DEGs) between the SCI group and the LV_SCI group, and 153 DEGs specific to LV_SCI between the (SCI vs LV_SCI) and (SCI vs CON) comparisons. The top 20 biological transition terms were identified by Gene ontology (GO) analysis. The Kyoto Gene and Genomic Encyclopedia (KEGG) analysis showed that the LV_SCI group significantly upregulated the cholinergic synaptic & nicotine addiction and the neuroactive ligand receptor interaction signaling pathway. Enriched chord analysis analyzes key genes. Further cluster analysis, gene and protein interaction network analysis and RT-qPCR results showed that *Chrm2* and *Chrnb2* together significantly in both pathways. The proliferation of muscarinic cholinergic receptor subtype 2 (Chrm2) and nicotinic cholinergic receptor subtype β2 (Chrnb2), and the neurogenesis were elevated in the injury site of LV_SCI group by immunofluorescence. Further by subcellular localization, the LV_SCI group enhanced the expression of Chrnb2 at the cell membrane.

**Conclusion:**

Knockdown of α-Syn after SCI enhance motor function and promote neurogenesis probably through enhancing cholinergic signaling pathways and neuroreceptor interactions. This study not only further clarifies the understanding of the mechanism of knockdown of α-Syn on SCI but also helps to guide the treatment strategy for SCI.

## Background

Spinal cord injury (SCI) is defined as damage to the structure and function of the spinal cord due to various causes, resulting in movement, sensation dysfunction below the level of injury. The incidence and the disability rate are higher, as well as the clinical refractory is greater in the upper segment than the lower segment of SCI [1]. In addition, there are severe autonomic dysfunctions in the SCI of the thoracic segment 6 or above [2]. Primary lesions include physical trauma of white matter and gray matter and vascular system collapse followed by secondary lesions, such as demyelination, peripheral immune cell infiltration, impaired neurotransmission, and neuronal apoptosis [3]. From the destruction of tissue structure, pathophysiological changes and abnormalities of signaling pathways, the release of a large number of cytokines, proteins and metabolites may be an important factor influencing prognosis. Over the years, SCI research has been dedicated to promoting long-distance growth of CNS motor axons. The main research focus is secondary lesions, which are investigated through such methods as analysis of the regulation of the immune response [3], induction of endogenous neural stem cells [4], and neurotrophic modulators to observe changes in neurogenesis and motor function [5].

In recent years, and studies have shown that endogenous α-synuclein (α-Syn) is involved in many pathophysiological processes in the secondary injury stage after acute SCI [6]. *Snca* is a key gene that encode α-Syn. Intracellular aggregation of α-Syn induces dopamine neuronal death, many α-Syn pathogenic features take precedence over motor dysfunction with non-motor function autonomic dysfunction, such as Parkinson’s disease (PD), multiple system atrophy (MSA) [6, 7]. In vitro and in vivo experiments have reported that inhibition of α-Syn expression can reduce neuroinflammation, increase neurotrophic factor expression, inhibit apoptosis, and promote nerve regeneration [8, 9]. For the production, aggregation or downstream effects of α-Syn may have strong potential for reducing the devastating complications of SCI in patients and may become the focus of future research. Nevertheless, the mechanism governing the function of α-Syn has not been determined to date.

Next-generation sequencing (NGS) has become a common technique in biology. RNA sequencing (RNA-seq) is one of the most complex applications of NGS and is a technique for sequencing and aligning RNA horizontal sequences to obtain transcriptome information. Because this method has higher accuracy than gene microarray analysis, it has higher sensitivity for detecting low-expression genes and less demand for a priori biological information. In addition, RNA-seq has significant advantages, such as low cost, high sensitivity, high throughput and good reproducibility. Both known and unknown transcripts can be detected by the RNA-seq method [10]. In view of the pathogenic characteristics of α-Syn, for the first time, in this study, we obtained differentially expressed genes (DEGs) by comparing the T3-SCI group (SCI group) and the lentiviral vector-mediated knockdown *Snca* (LV_SCI group) after T3-SCI. Compared with the sham operation group (CON group), the DEGs unique to the LV_SCI group were compared for bioinformatics analysis. This study may provide new clues for studying the mechanism of SCI and provide new molecular targets for the clinical treatment of SCI.

## Results

### Experimental procedure and animal recovery

**Fig. 1:**
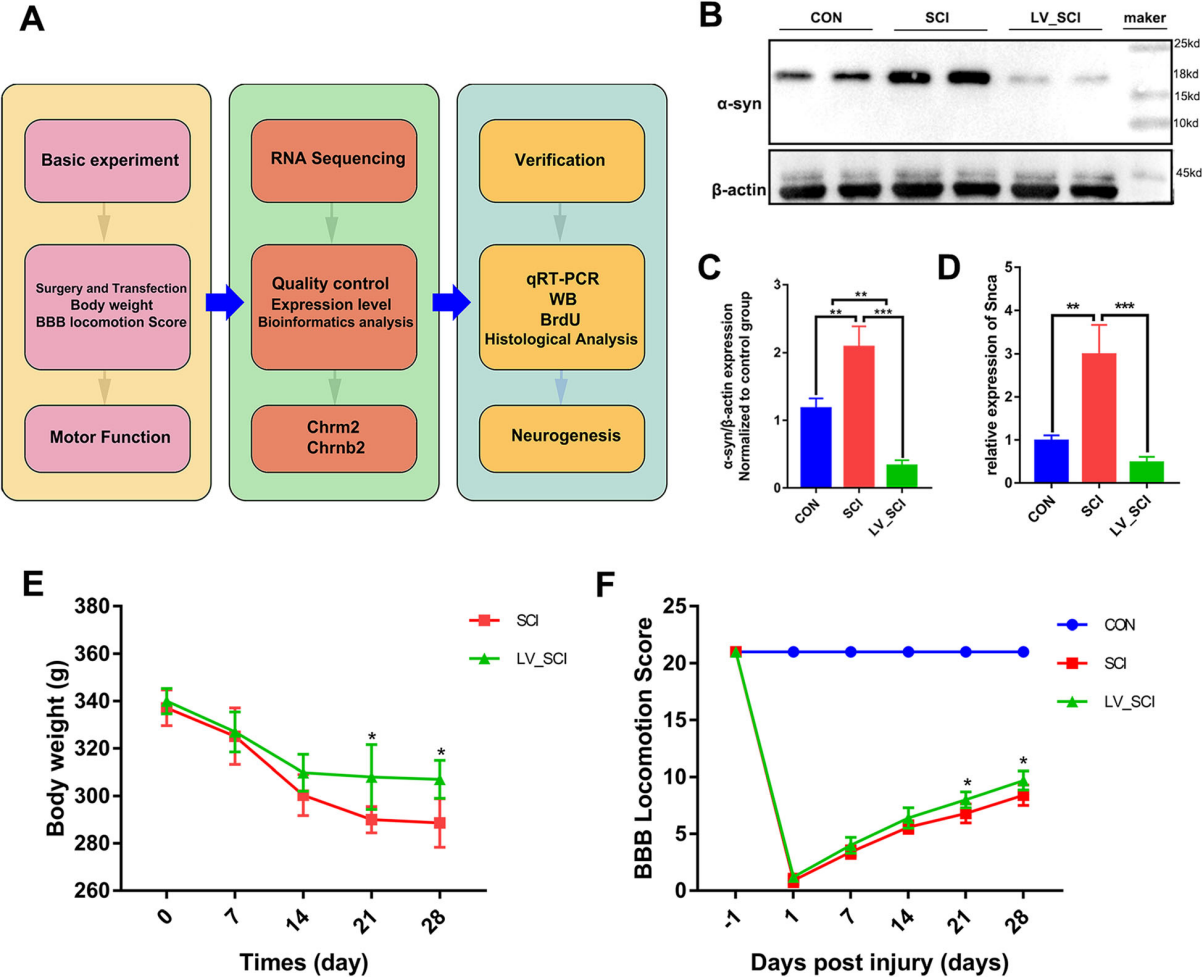
Experimental protocol and animal recovery. **a** Details of the experimental protocol. **b** Representative Western blotting results of α-Syn between the three groups 28 days after SCI. **c** Quantification of Western blotting data from B. *n* = 3. **d** Relative expression levels of *Snca* by qRT-PCR. *n* = 3. **e** Changes in body weight of rats. *n* = 5. **f** Quantification of the BBB Scale score on day 28 of each group. *n* = 5. *p < 0.05. * *p* < 0.05, ** *p* < 0.01, ***p < 0.001

The entire experimental procedure is shown in Fig. 1a. We first verified the expression of α-Syn in the injury site on the 28th day after SCI was higher than that in the CON group, as well as was significantly downregulated by shRNA silencing α-Syn (LV_SCI group), as shown in Fig. 1b, c. Also, the mRNA expression of *Snca* was examined by qPCR (Fig. 1d), and the mRNA level of *Snca* in LV_SCI group was significantly lower than SCI group (*P* = 0.0007). We examined the BBB exercise score throughout the experiment. The changes in rat body weight and BBB exercise score over time are shown in Fig. 1e, f. Body weight was higher in the LV_SCI group than in the SCI group on day 28, and the BBB exercise score was higher in the LV_SCI group than in the SCI group on days 21 and 28, and the scores in the two groups were significantly lower than those in the CON group. It was shown that administration of LV-*Snca*-shRNA can improve the functional recovery of BBB in rodents after severe T3 contusion.

### Differentially expressed mRNA

**Fig. 2:**
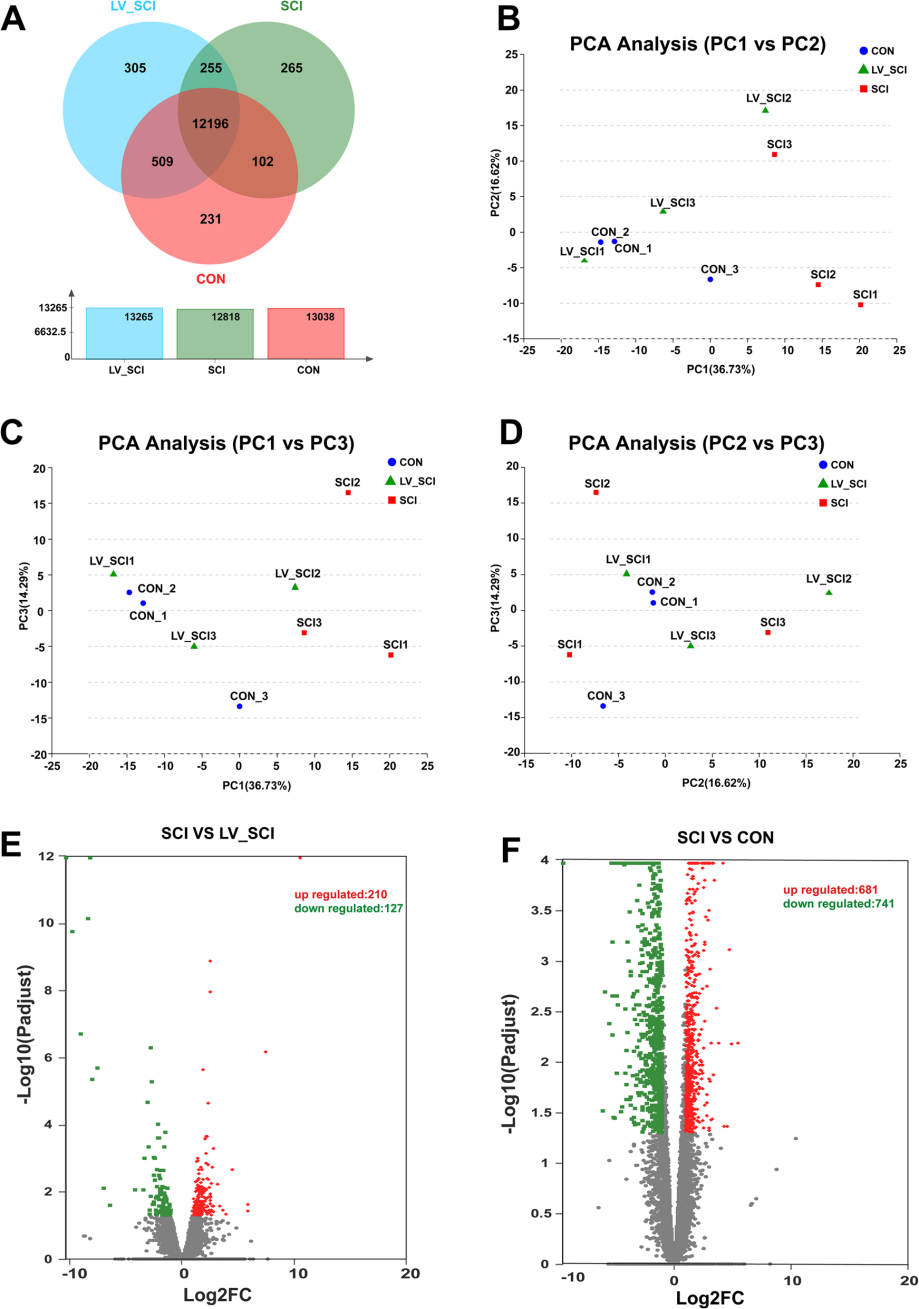
Changes in mRNA expression profiles between groups. **a** The amount of expression of the three groups in the entire transcriptome. **b**-**d** Principal component analysis (PCA) score plot with the two principal components (PC1vs PC2, PC2vs PC3, and PC1vs PC3). **e** Volcano plots show up- and down-regulated mRNA transcripts in the SCI vs LV_SCI group. **f** Volcano plots indicate up- and down-regulated mRNA transcripts in the CON vs SCI group

To further understand the role and mechanism of knockdown of α-Syn on SCI, three sets of biological replicates were established in each group for RNA-seq. A summary of sequence assembly after sequencing of Illumina is presented in Additional file 1: Table S1. The Q30 basic mass fraction of the sample exceeded 90.06%, meeting the requirements of subsequent analysis. The utilization of the mapping between the transcriptome and the reference genome results in clean readings for further analysis (Additional file 2: Table S2). In the RNA-seq results, 32,883 genes were detected, and Venn analysis between samples showed the amount of expressed genes in each group (Fig. 2a). We first characterised differences between the gene expression profiles of 9 samples, utilising an unsupervised classification method – Principal Component Analysis (PCA). Figure 2b-d revealed that samples of three biological replicates could be assigned to three groups, referred to as CON1–3, SCI1–3 and LV_SCI1–3. The PCA model revealed that the largest variation of the PC1 could explain 36.73% of the variation while principal component 2 and 3 explained 16.62 and 14.29%, respectively [11]. In Fig. 2e, the SCI and LV_SCI group differential gene volcano maps showed that 337 genes were differentially expressed between the two groups, of which 210 genes were upregulated and 127 genes were downregulated (DEGs listed in **A**dditional file 3: Table S3 and Additional file 4: Table S4). Figure 2f shows the volcano map of the differential gene between SCI and CON. The results showed that 1422 genes were differentially expressed between the two groups, of which 681 genes were upregulated and 741 genes were downregulated.

### GO function enrichment analysis

**Fig. 3:**
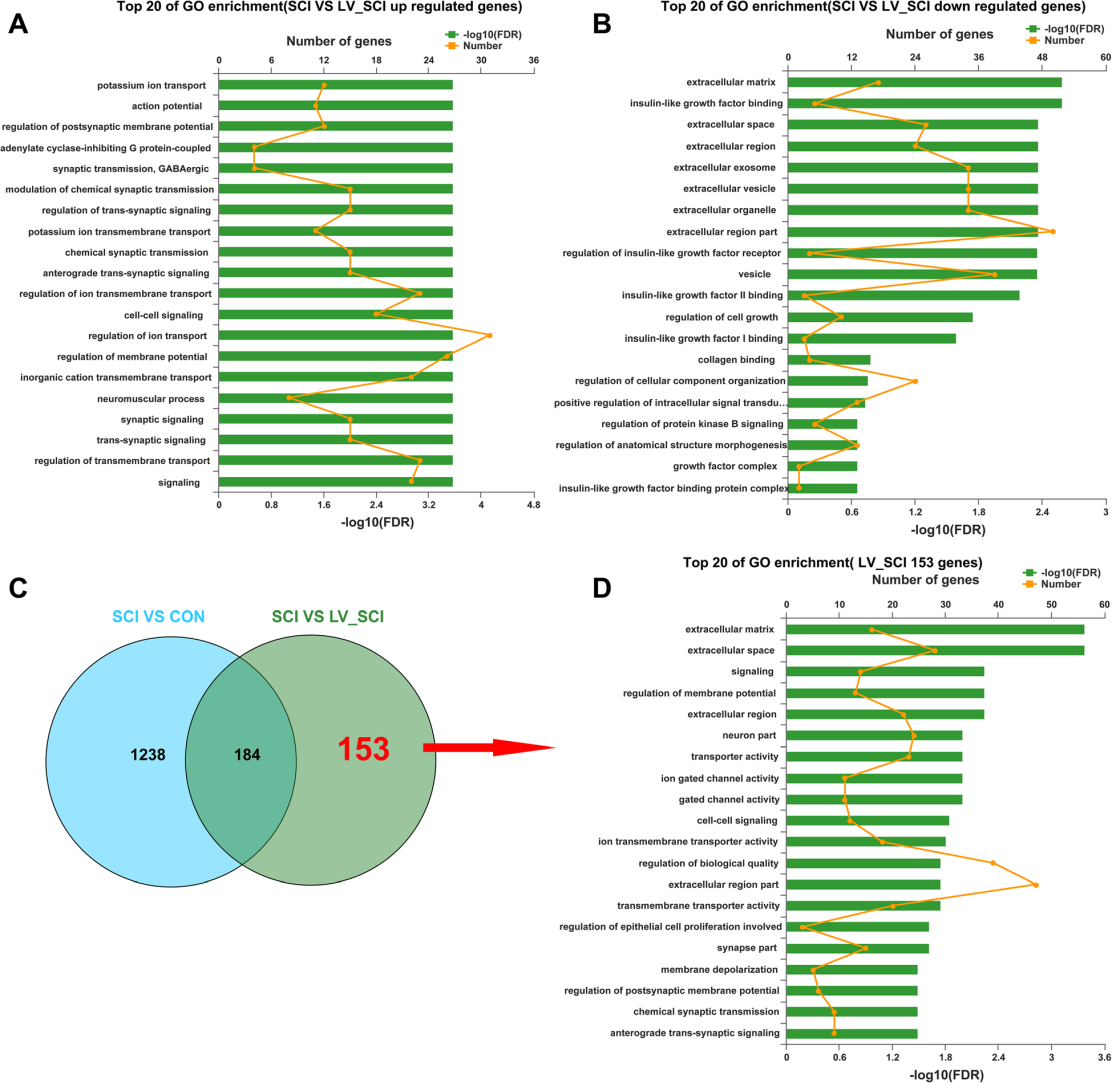
GO Enrichment Analysis of DEGs. **a** GO enrichment histogram of SCI and LV_SCI group upregulated DEGs. **b** GO enrichment histogram of SCI and LV_SCI group downregulated DEGs. **c** Venn diagram between the SCI vs CON and SCI vs LV_SCI. **d** Compared to SCI and CON group, the GO enrichment histogram of LV_SCI group DEGs. The ordinate represents the GO term, the upper abscissa indicates the number of genes in the GO term, and the lower abscissa indicates the level of significance of the enrichment

To further explore the effect of knocking down α-Syn on the regulation of SCI, the GO database was used to analyze the function set information between the groups, and the significance of the function set to the differentially expressed genes was calculated. Figure 3a shows the enrichment results of the TOP 20 GO enrichment display the 210 DEGs between the SCI group and the LV_SCI group based on the DEG-based biological process (BP) category (Additional file 5: Table S5). Among these GO enrichment, the “potassium ion transport”,“ regulation of postsynaptic membrane potential “,“ adenylate cyclase “,“ GABAergic synaptic transmission”, other transmembrane transport or synaptic transmission enrichment rates are the highest. Similarly, Fig. 3b shows that 127 downregulated DEGs in the LV_SCI group for GO enrichment, with outer membrane transport of “extracellular matrix”, “insulin-like growth factor signaling pathway”, “extracellular space”, “extracellular region” and synaptic transmission or other regulation have the highest rate of enrichment (Additional file 6:Tables S6). The Venn analysis of Fig. 3c demonstrates the regulation of 153 DEGs specific to LV_SCI between the (SCI vs LV_SCI) and (SCI vs CON) comparisons (Additional file 7: Table S7), while GO enrichment of its 153 DEGs (Fig. 3d). The highly enriched have “modulation of membrane potential “,“ cell-cell signaling “and “biomass regulation “. Indeed, the GO analysis (Fig. 3b and d) indicates that the “extracellular matrix”, “extracellular space”, “extracellular region” are the most highly represented in terms of both -log10(FDR) and number of genes. We performed a visual cluster analysis of 41 genes in the three GO enrichment projects; further, the STRING database (http://string-db.org/) was used for protein-protein interaction (PPI) analysis to find the central node gene (Additional file 8: Figure S1), including collagen, type I, alpha 2 (*Col1a2*); elastin (*Eln*); decorin (*Dcn*); insulin-like growth factor 2 (*Igf2*), because these down-regulated genes may affect the outcome of SCI. Taken together, the results of this analysis indicate that knocking down α-Syn primarily affects transmembrane transport, synaptic transmission of neurotransmitters -related biological processes.

### KEGG pathway analysis

**Fig. 4:**
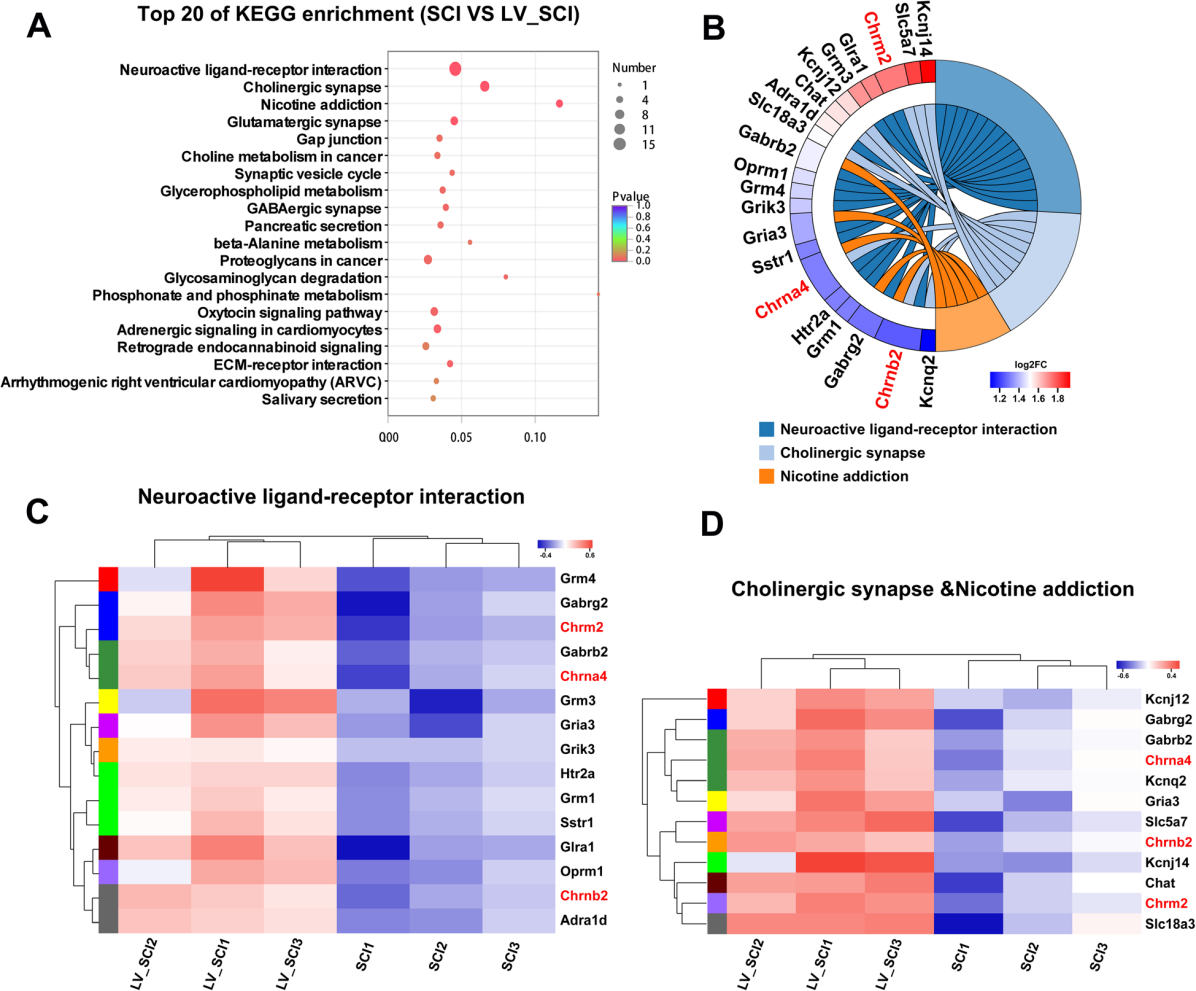
Analysis of differential gene KEGG pathway enrichment. **a** KEGG pathway enrichment analysis of DEGs. **b** The KEGG pathway of DEGs enriches the chord diagram. **c**, **d** Cluster Analysis of KEGG-enriched DEGs

The KEGG pathway enrichment assay further determines the signaling pathways involved in DEGs. The bubble map of the differential gene provides a graphical representation of the top 20 most enriched pathways of DEGs (Fig. 4a). Enriched KEGG pathways from the differentially expressed genes are listed in Additional file 9: Table S8**.** Among the many enriched signaling pathways, the cholinergic synapse & nicotine addiction and neuroactive ligand-receptor interaction were the most significant. Further enrichment chord analysis (Fig. 4b) showed that significantly upregulated cholinergic receptor muscarinic subtype 2 (*Chrm2*) and cholinergic receptor nicotinic subtype α4β2 (*Chrnb2, Chrna4*) were involved in both conduction pathways. Nicotine addiction pathway belongs to the class of nicotine type of cholinergic synapse, so we consider it as a large category for further analysis. At the same time, further clustering analysis was performed on the two differential signaling pathways (Fig. 4c, d).

**Fig. 5:**
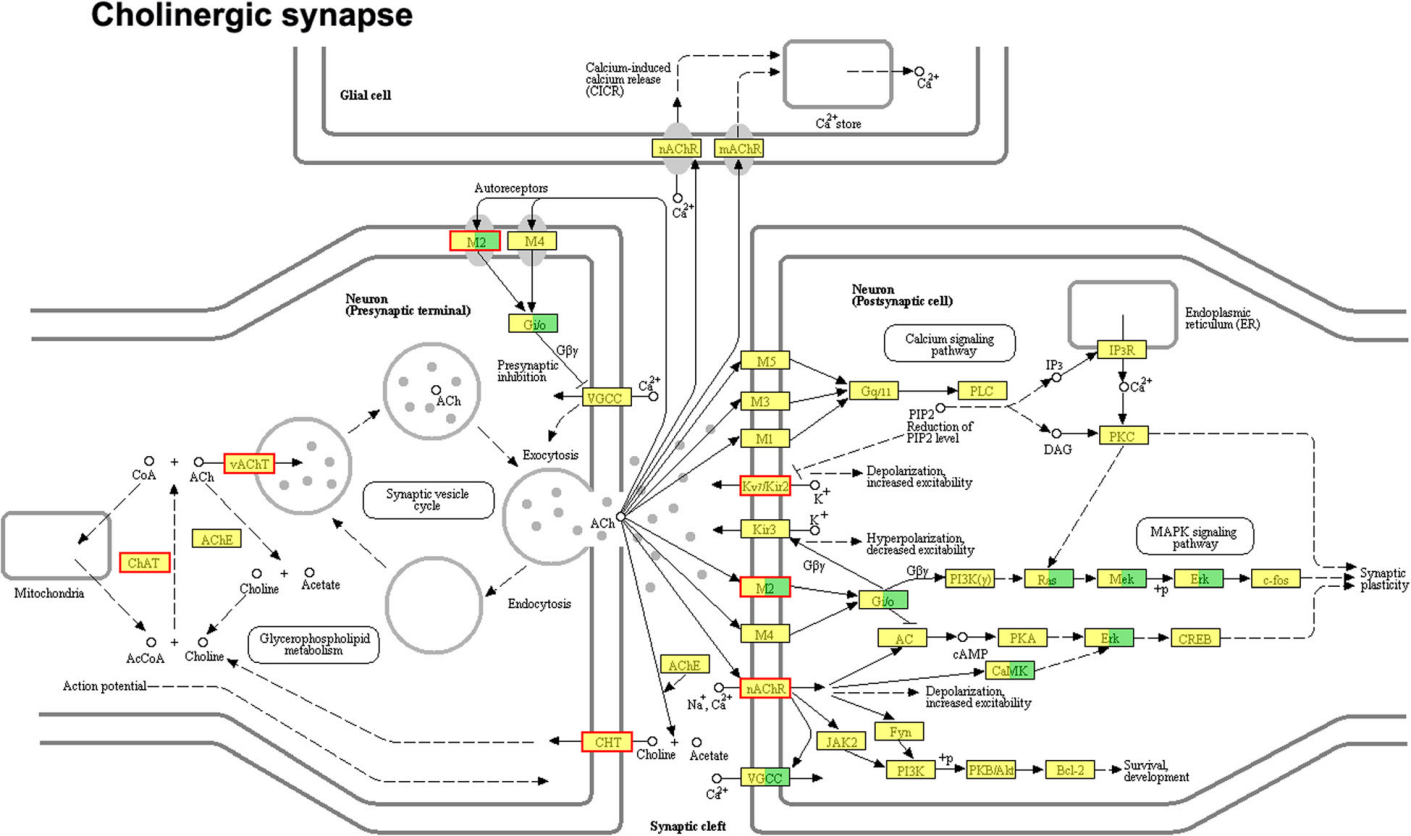
KEGG pathway map of cholinergic synapse

**Fig. 6:**
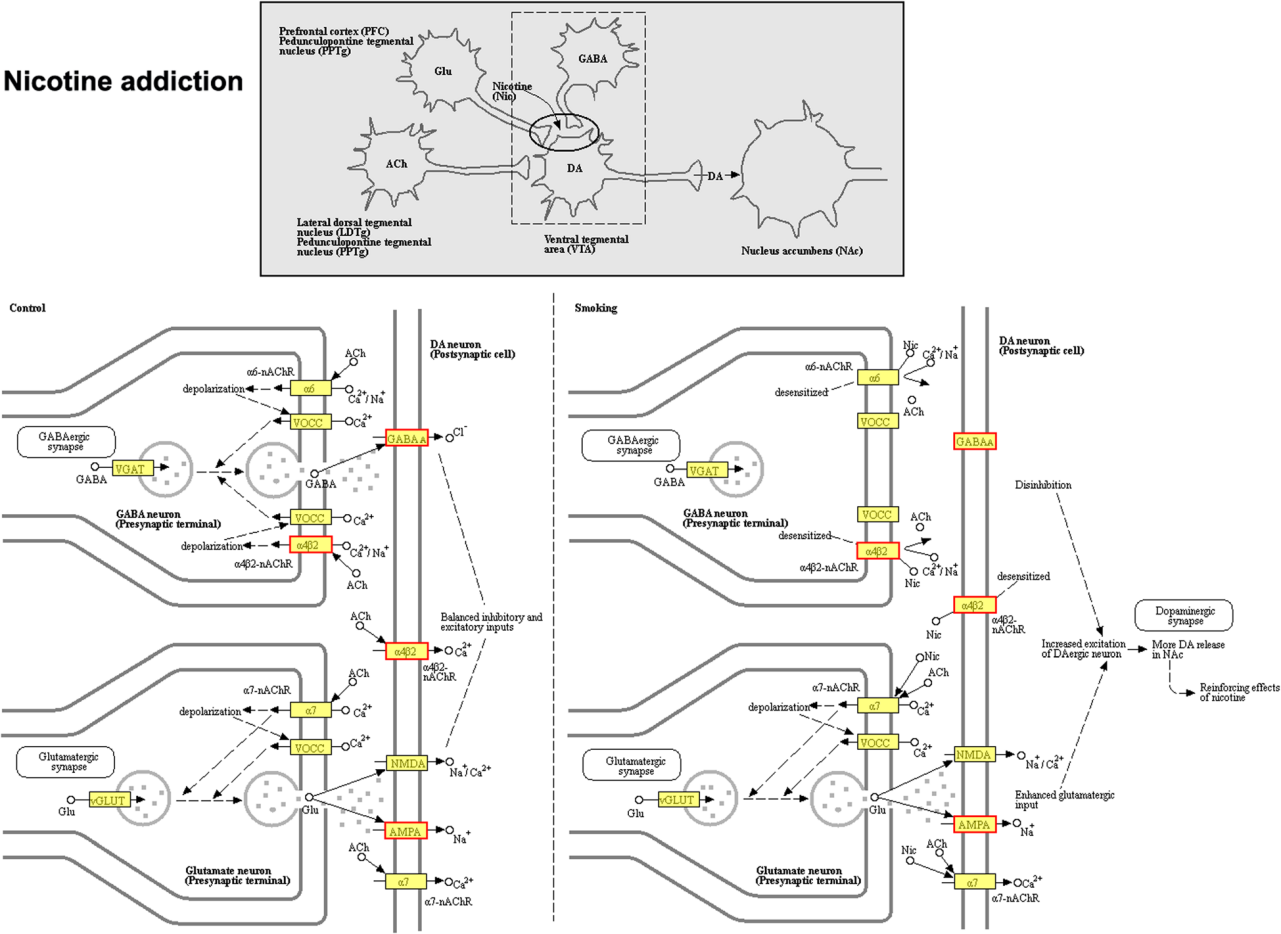
KEGG pathway map of nicotine addiction

The differential genes involved in the cholinergic synapse & nicotine addiction and neuroactive ligand-receptor interaction pathway include solute carrier family: *Slc5a7*, *Slc18a3*; potassium voltage-gated channel subfamily: *Kcnj14, Kcnj12, Kcnq2*; cholinergic receptor: *Chrm2, Chrnb2, Chat, Chrna4*; glutamate metabotropic receptor: *Grm3, Grm1, Grm4*; glutamate ionotropic receptor: *Grik3, Gria3*; gamma-aminobutyric acid type A receptor: *Gabrg2, Gabrb2*; glycine receptor: *Glra1*; somatostatin receptor: *Sstr1*; opioid receptor: *Oprm1*; 5-hydroxytryptamine receptor: *Htr2a*; adrenoceptor: *Adra1d*. All 21 differentially expressed genes are listed in Additional file 10: Table S9 for reference. Figure 5 and 6 show KEGG pathway map plots for cholinergic synapse & nicotine addiction, the red boxed gene in the figure indicates the differentially expressed genes involved in this pathway, and all products with a colored background in the figure belong to the sequencing gene/transcript KEGG background annotation results. KEGG mapping of cholinergic signaling pathways and neuroreceptor interactions pathways showed in Additional file 11: Figure S2. At the same time, compared with the SCI group, KEGG analysis of the downregulated genes in the LV_SCI group suggested multiple apoptosis and inflammatory pathways, although there was no statistical difference (Additional file 12: Table S10).

In addition, we analyzed the upregulated DEGs in the SCI group compared to the CON group, mainly “TNF signaling pathway”, “cytokine-cytokine receptor interaction”, “antigen processing and presentation”, “apoptosis” and other pathways (Additional file 13: Table S11). Compared with the CON group, the downregulated DEGs in the SCI group are mainly “neuroactive ligand-receptor interaction”, “glutamatergic synapse”, “circadian entrainment”, “retrograde endocannabinoid signaling” (Additional file 14: Table S12 and Additional file 15: Figure S3).

### Gene and protein interaction network analysis

**Fig. 7:**
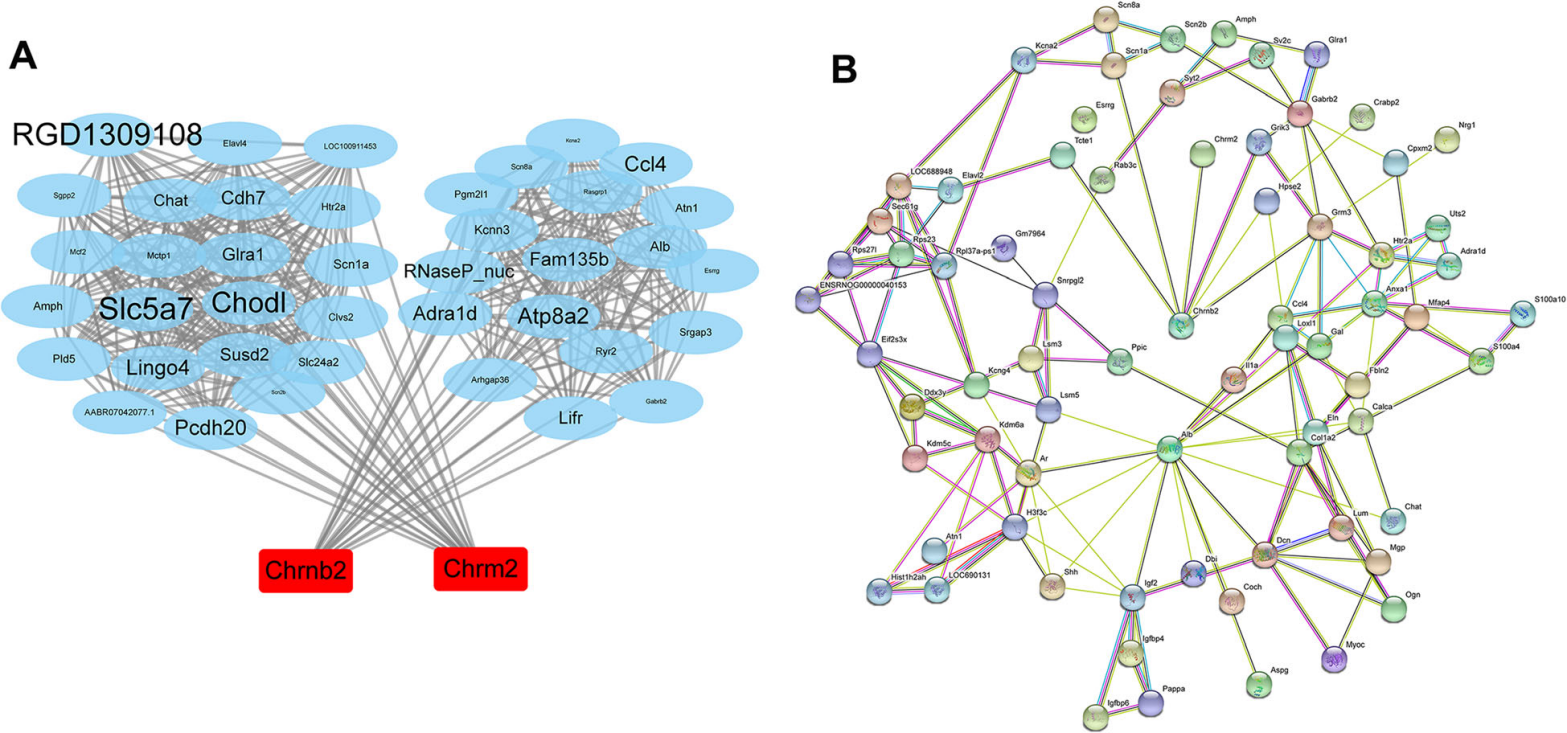
Analysis of differential gene and protein interaction network. (**a**) Interplay analysis of expression-related genes with Chrm2 and Chrnb2 as genes of interest. (**b**) Protein interaction of DEGs between SCI and LV_SCI group

*Chrm2* and *Chrnb2* were used as genes of interest to analyze the correlation between gene expression, and the correlation coefficient between gene and gene was obtained using the Spearman correlation algorithm and then mapped into a visualization network using Cytoscape software (Fig. 7a). The internode connection represents that there is a correlation between gene expression, and the larger the node is, the stronger the correlation is between the gene and the expression of other genes. The differential genes with high correlation with *Chrm2* and *Chrnb2* are solute carrier family: *Slc5a7, Slc24a2*; neurotransmitter receptor and transport related genes: *Atp8a2, Chat, Htr2a, Adra1d, Glra1*; ion channel: *Kcnn3, Scn1a*; adhesion protein: *Cdh7, Chodl, Pcdh20, Alb*; cytokine: *Ccl4, Lifr, Mcf2*. Network analysis of protein proteins interacting (PPI) with genes such as *Chrnb2* and *Chrm2* using the STRING database, the SCI group and the LV_SCI group’s DEGs are mapped to the interaction relationship for network construction, and the key nodes in the interaction network are obtained according to the intergene connectivity and other indicators (Fig. 7b). The results showed that *Chrnb2* interacted directly with *Chrm2*, and the relative expression of the *Chrnb2* gene has a higher degree of centrality (DC), indicating that this gene is more strongly associated with other proteins. This gene can be speculated to play a more important role in the LV_SCI group.

### RT-qPCR verification of RNA-seq

**Fig. 8:**
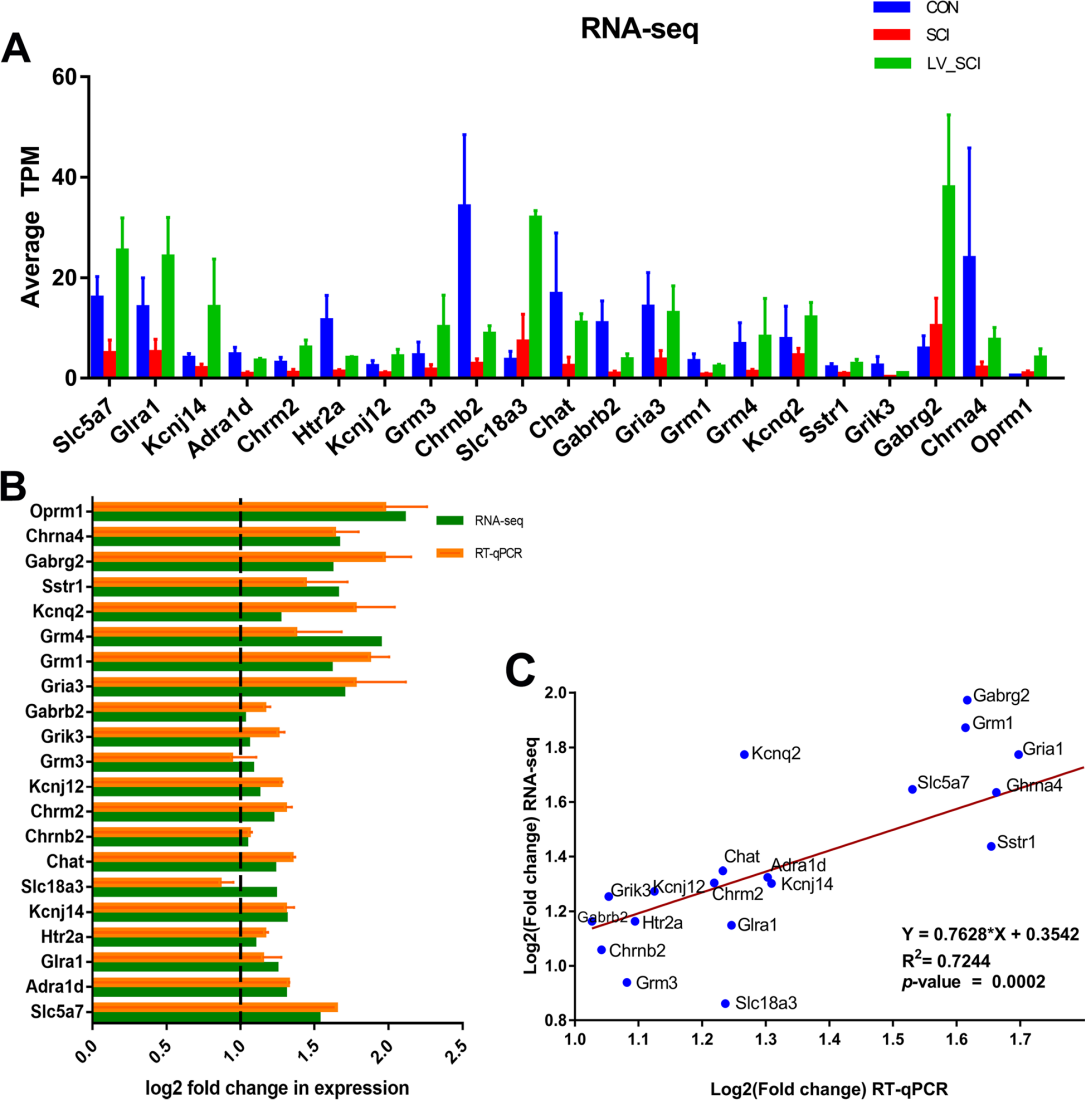
qRT-PCR validation of RNA-seq. **a** Average TPM values of 21 DEGs obtained in RNA-seq. **b** Comparison of transcript expression in terms of fold change was measured by RNA-Seq and qPCR. **c** Correlation analysis of qRT-PCR and RNA-seq

Figure 8a shows TPM changes of 21 DEGs in RNA-seq. To further validate the transcriptome sequencing results of DEGs, 21 differentially expressed mRNA from cholinergic synapse & nicotine addiction and neuroactive ligand-receptor interaction pathways was verified by RT-qPCR (Fig. 8a). *Gapdh* is used as an internal reference. (Fig. 8b). Correlation analysis was performed based on the Log2FoldChange value of DEGs, and the correlation coefficient was 0.7224 (*P* = 0.0002) (Fig. 8c).

### Knockdown of α-Syn promotes the proliferation of Chrm2 and Chrnb2 cells at the injury site

**Fig. 9:**
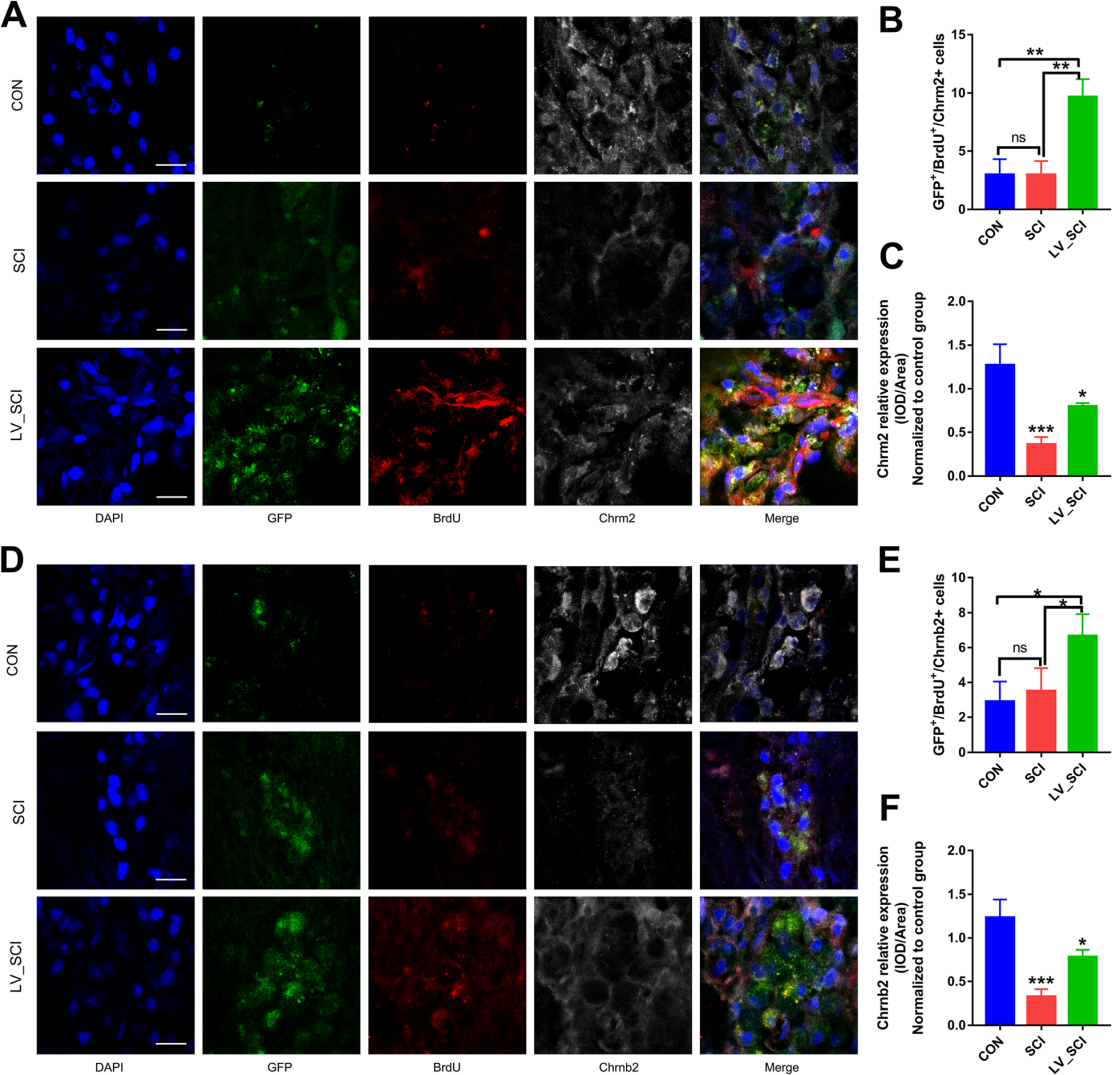
Cell proliferation and histological analysis of Chrm2 and Chrnb2 at the injury site. **a** Representative images showing GFP+/BrdU+/Chrm2+ triple staining by immunofluorescence. Scale bar = 35 μm. **b**, **c** Quantitative analysis results from Fig. **a**, **b**. *n* = 3. * = *p* < 0.05. **d** Representative images showing GFP+/BrdU+/Chrnb2+ triple staining by immunofluorescence. Scale bar = 35 μm. **e**, **f** Quantitative analysis results from Fig. **a**, **b**. *n* = 3. *p < 0.05. * *p* < 0.05, ** *p* < 0.01, ***p < 0.001

The proliferation of Chrm2 and Chrnb2 positive cells in the SCI site of rats was evaluated by 5-bromodeoxyuridine (BrdU) labeling to examine whether knockdown of α-Syn was induced by Chrm2 and Chrnb2 cells. GFP was transduced with a lentiviral vector expressing the green fluorescence protein, the SCI group was transduced with a blank vector, the LV_SCI group was transduced with a lentiviral vector expressing the target gene *Snca*-shRNA, and GFP signal indicates knockout of α-Syn at this site. As shown in Fig. 9, the CON group highly expressed Chrm2 and Chrnb2 relative to the SCI and LV_SCI groups, indicates that there is a large amount of cholinergic receptors in the intact T3 spinal cord. However, the number of Chrm2 + BrdU+ and Chrnb2 + BrdU+ double positive cells was significantly increased in LV_SCI rats compared to the CON and SCI rats (Fig. 9b, e). Compared with the SCI group, the LV_SCI group showed new Chrm2 and Chrnb2 cell proliferation at the injury site (Fig. 9c, f), it indicated that silencing α-Syn promoted the production of cholinergic receptors involved in neurotransmitter transmission, further confirmed the increased expression of Chrm2 and Chrnb2.

### Effect of α-Syn knockdown on SCI neurogenesis and Chrm2 and Chrnb2 subcellular localization

**Fig. 10:**
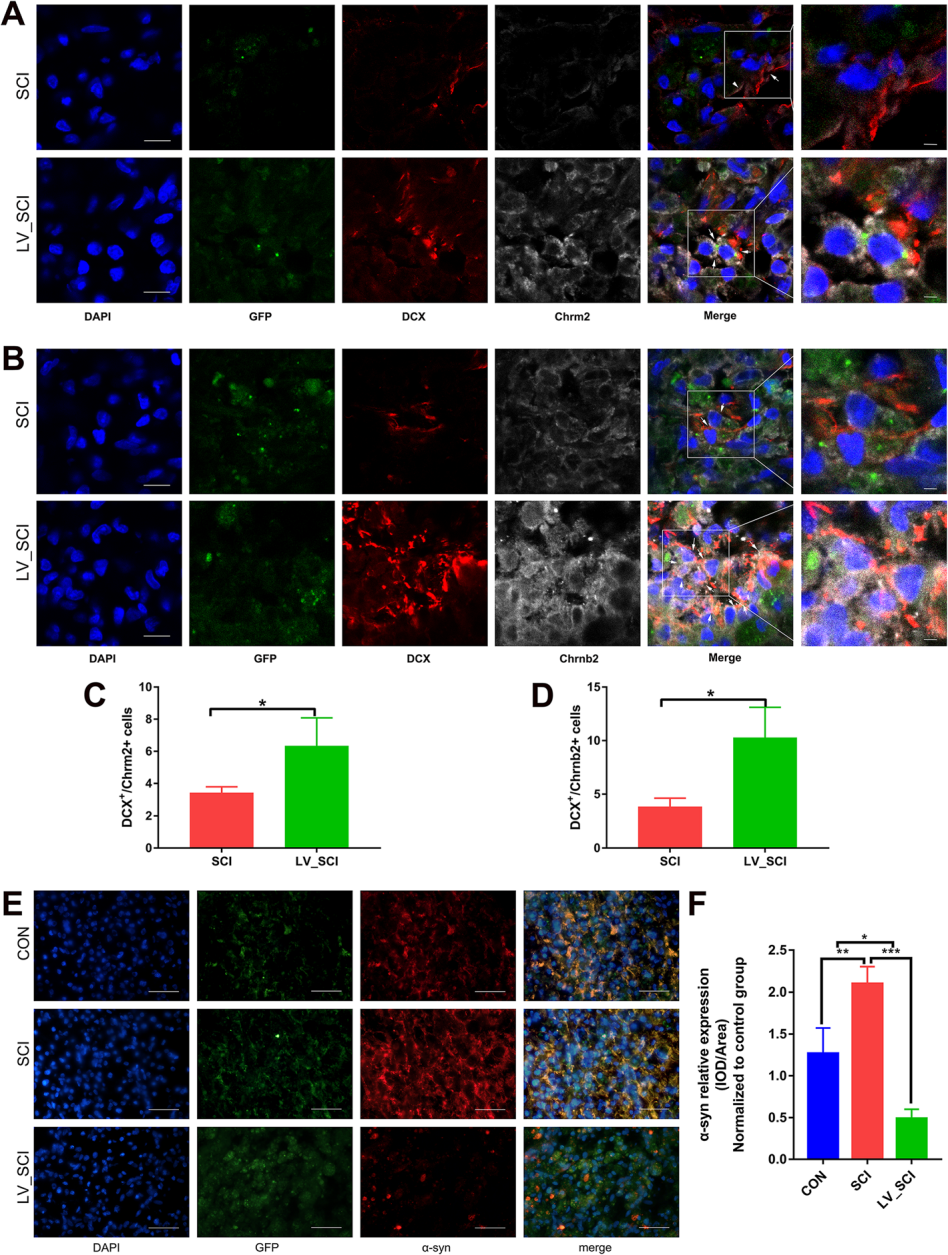
Neurogenesis and subcellular localization after spinal cord injury. **a** Representative images showing GFP+/BrdU+/Chrm2+ triple staining by immunofluorescence, Scale bar = 35 μm. **b** Representative images showing GFP+/BrdU+/Chrnb2+ triple staining by immunofluorescence. Scale bar = 35 μm. **c-d** Quantitative analysis results from Fig. **a**, **b**. *n* = 3. * = *p* < 0.05. **e** Representative images showing GFP+/α-Syn + double staining by immunofluorescence. **f** Quantitative analysis results from Fig. **e**, *n* = 3. *p < 0.05. * *p* < 0.05, ** *p* < 0.01, ***p < 0.001

To further investigate the mechanism of action of the cholinergic pathway in SCI, we determined the expression of Chrm2 and Chrnb2 in neurogenesis and their subcellular localization. Doublecortin (DCX) antibodies can be used to specifically label new neurons [12]. Under CNS injury state, DCX promotes the differentiation and maturation of new neural stem cells as well as promotes their migration from the origin to the lesion or damaged area to replace neurons. Therefore, DCX should be regarded as the gold standard marker for detecting neurogenesis [13]. We performed multiple immunofluorescence staining on Chrm2, Chrnb2 and DCX in the injury sites of the SCI group and LV_SCI group. In Fig. 10a, b, we observed that the number of DCX+ Chrm2+ and DCX+ Chrnb2+ double-positive cells in the LV_SCI group was significantly higher than that in the SCI group (Fig. 8c, d), further indicating that the cholinergic receptors increased by silencing α-Syn at the injury site promoted neonatal regeneration. DCX staining was expressed as filamentous protein, cytoplasmic expression marked the neurite state. Both Chrm2 and Chrnb2 proteins are expressed in the cell membrane and membrane peripheral-secreted proteins. In addition, we used α-Syn specific antibody markers to detect whether α-Syn was knocked down. In Fig. 10e, the α-Syn of LV_SCI rats was significantly lower (*P* < 0.001) than SCI rats, while the SCI group was significantly higher than that of the CON group (Fig. 10f). All immunofluorescence figures were from the injury site of spinal cord.

## Discussions

Severe and often irreversible defects in spinal cord injury lead to reduced sensorimotor and autonomic function. The secondary injury process of SCI will worsen the neurological outcome, regardless of whether it is organic or functional; therefore, treating this stage should be an important goal of therapy [14]. α-Syn is a CNS-enriched protein. An increasing number of studies have reported that α-Syn affects the neurological prognosis in secondary nerve injury. Intervention by various means prevents the death of neurons and promotes functional recovery. In the past several years, there has been evidence that misfolded α-Syn, the major component of the Lewy body, accumulates in the brains of patients with Parkinson’s disease (PD) and traumatic CNS-related diseases [15]. We found that α-Syn expression was elevated after SCI, and α-Syn was downregulated by transfection of lentiviral *Snca*-shRNA lesions. This study newly found that silencing α-Syn activates the cholinergic pathway of the spinal cord after SCI as well as promotes neural stem cell production.

We used RNA-Seq analysis as an unbiased method [16] to screen for differentially expressed genes in SCI tissues from surgically modified and knockdown rats in the α-Syn group 28 days after surgery. A total of 337 differentially expressed genes, including 210 upregulated genes and 127 downregulated genes, were screened. It is worth pondering that the GO analysis of 127 downregulated DEGs indicates that the “extracellular matrix”, “extracellular space”, “extracellular region” and other similar categories are the most highly enriched, wherein the elastin and collagen related genes are down-regulated. The extracellular matrix is a complex 3-dimensional structural framework, and the components of the extracellular matrix have signal transduction and regulation in the fate and function of neurons and non-neuronal cells in the CNS. Collagen, fibronectin and laminin are involved in wound healing and regeneration and are therefore the main focus of current SCI treatment [17]. However, glial scars formed around the damaged area after SCI, and the type of collagen protein are also a major contributor to scar formation. The most famous role of scar tissue hinders the growth of axons [17]. Extracellular matrix components may affect the aggregation of α-Syn [18]. This study suggests that α-Syn silencing may alter the extracellular matrix, and that changes may affect the prognosis of SCI [19]. KEGG enrichment screened two pathways that are distinctly different and closely related to pathophysiological processes, namely, the cholinergic synapse & nicotine addiction and the neuroactive ligand-receptor interaction. The 21 DEGs in the pathway screened two closely related genes, *Chrm2* and *Chrnb2*.Interestingly, RT-qPCR verification, cell proliferation and functional verification revealed that *Chrm2* and *Chrnb2* play an important role in neurogenesis.

SCI causes severe disruption of the neuronal circuit, leading to motor function defects. Cholinergic dysfunction has been a familiar topic for many years to researchers of SCI [20]. Few studies have been devoted to the possible reasons of the selective degeneration of cholinergic neurons in accumulation of α-Syn. What are the possible connections between α-Syn and function of cholinergic systems? First, the characteristics of α-Syn aggregation in the CNS, it has been suggested that aggregation of α-Syn is preferentially formed in projection neurons with long, thin, unmyelinated axons [21]. The hyper branched axons of autonomic myelin are the structural basis for the accumulation of α-Syn. It is tended that energy/metabolic burden and oxidative stress can be significantly happened in these types of neurons, thereby aggravating secondary injury responses [21]. The autonomic nerve cells induced by aggregation of α-Syn significantly disturbs the normal neurotransmitter balance, which may be one of the main causes of secondary injury events. α-Syn is selectively expressed in the nervous system, in the distal gut, α-Syn is expressed in most of the intestinal cholinergic axons [22]. Second, how a-Syn aggregation causes cholinergic neuronal dysfunction. In the entire nervous system, α-Syn is concentrated in axons rather than in dendrites or neural cell bodies. Thus, it appears to play a role in the release or recirculation of synaptic vesicles in presynaptic terminals then makes autonomic neurons susceptible to degeneration [22]. α-Syn aggregation causes mitochondrial dysfunction and aggravates cellular oxidative stress [23, 24]; in turn, oxidative stress in injured neurons further promotes α-Syn transfer and spreads along the vagus nerve [25, 26]. Thus, it was shown that the loss of cholinergic neurons is associated with overexpression and accumulation of α-Syn and microglia activation [27]. α-Syn can interact with neuronal N-methyl-D-aspartate receptors for cholinergic dysfunction [28].

### Muscarinic cholinergic receptor (mAChR)

The muscarinic acetylcholine receptor (mAChR) is a G-protein coupled receptor that plays an important role in neurogenesis, the survival of newborn neurons and long-term potentiation (LTP). The major cholinergic receptor in the rat spinal cord is mAChR subtype 2 (Chrm2), which is mainly distributed in the C-bouton pool (a cholinergic C-type terminal buckle that regulate motoneurons) of anterior horn motoneurons (MNs), regulating MNs excitability [29]. The imbalance of Chrm2 distribution in the MNs after SCI leads to incoordination or clonic movement, Chrm2 is also present in glutamatergic synapses and γ-aminobutyric acid interneurons [30]. A similar report indicates that mAChR inhibitors are effective in the treatment of neurogenic bladder after SCI [31], possibly due to an imbalance in the distribution of muscarinic receptors in the interneurons that innervate excitatory muscles or antagonist muscles after SCI. Studies have also shown that the expression of the muscarinic M1 receptor gene in the SCI group shows a significant downregulation [32]. This newly study found that α-Syn silencing upregulates *Chrm2* after SCI. There are few studies on the regulation of muscarinic receptors by α-Syn, as well as the role between them is still unclear. Ettle B et al. found that the accumulation of α-Syn in MSA leads to the loss of myelin, the use of the muscarinic receptor inhibitor phenazine can reverse this myelin defect [33].

### Nicotinic cholinergic receptor (nAChR)

The major nAChR in CNS contains the subunit α4β2, which is present in presynaptic or postsynaptic and maintains various neuronal functions. The former regulate the release of acetylcholine or other neurotransmitters in the CNS, the latter is used to control interactions with metabolites and ionic gating. Our data suggest that most of the DEGs involved in cholinergic synapse & nicotine addiction are metabolic receptors and ion-gated families of neurotransmitters that may interact with nAChR to participate in synaptic plasticity. *Chrnb2* is the major DEG, interestingly, a decrease in the number of α4β2-nAChRs in PD patients indicates α4β2-nAChR-mediated cholinergic deficits in PD [34]. Interestingly, the number of α4β2-nAChRs in PD patients was reduced, indicating a cholinergic deficit mediated by α4β2-nAChR in PD. α4β2-nAChR is a sensitive target for the regulation of cholinergic signaling induced by oligomeric α-Syn [35].

Many studies have shown that the β2 subunit of the nAChR plays an important role in regulating cell proliferation in neurogenesis. Among them, enhancement of α4β2-nAChR can inhibit chemotherapeutic drug-induced cognitive disorders and also promote hippocampal neurogenesis [36]. The use of donepezil, an acetylcholinesterase inhibitor, in many neurodegenerative lesions can enhance hippocampal neurogenesis [37]. DCX, an essential factor in the differentiation and migration of newborn neurons, is used as a marker to detect the process of neural recruitment. In this study, immunofluorescence experiments confirmed that silencing α-Syn mainly upregulates muscarinic or nicotinic cholinergic receptors to promote DCX production, promote nerve regeneration at the injury site as well as improve motor function [13]. In summary, the mechanisms of Chrm2 and Chrm2 in this study complement each other. We hypothesize that the Chrm2 emphasizes the balance of neurotransmitters, while the Chrnb2 promote neurogenesis, the Chrm2 and Chrm2 together achieve the effectiveness of neurological function.

This study also presented that a large number of DEGs in the SCI group significantly upregulated multiple inflammatory or apoptotic pathways compared with the CON rats, such as “TNF signaling pathway”, “cytokine-cytokine receptor interaction”, “antigen processing and presentation”, “apoptosis” and other pathways. However, the pathways of inflammation or apoptosis were slightly downregulated after silencing α-Syn, although there was no statistical difference. It may be possible to provide a new direction for previous studies on the mechanism by which knockdown of α-Syn can inhibit apoptosis and promote nerve regeneration after SCI. In the study of traumatic CNS injuries, another crucial role of the cholinergic pathway is the “choline anti-inflammatory reflex pathway” [38]. Studies have found that vagus nerve stimulation (VNS) causes a significant decrease in the systemic inflammatory response by endotoxin in experimental animals. This effect is mediated by acetylcholine (ACh), which stimulates nicotinic receptors on spleen macrophages [39]. The immune and nervous systems are tightly bound, and each system can affect another system in response to an in vivo balanced infection or inflammatory disturbance. Both microglia and astrocytes express cholinergic receptors [40]; therefore, it is possible to produce some cholinergic effects through these non-neuronal cells [41]. Activation of cholinergic receptors reduces the release of tumor necrosis factor-α (TNF-α), interleukin-1β (IL-1β) and interleukin-6 (IL-6) [31]. It is possible that ACh released from immune cells or cholinergic neurons regulates immune function by acting on its receptor in an autocrine or paracrine manner [42]. Knocking out α-Syn promotes proliferation of cholinergic neuron receptor cells, thereby promoting the “cholinergic anti-inflammatory pathway” to regulate neuroinflammation and reduce secondary reactions of SCI. At the same time, Jiang et al. [43] found that the use of nicotine inhibits the infiltration of CCR2 + Ly6C high pro-inflammatory monocytes and neutrophils into the CNS by activating nicotinic AChRs in a mouse model, and the reduced pro-inflammatory cytokines may be a decrease in the number of M1 macrophages regulated by the cholinergic anti-inflammatory pathway [43].

Through GO analysis, 210 DEGs was mainly enriched in “potassium transport “,“regulatory post-synaptic membrane potential regulation “,“glutamate receptor signaling pathway “,"GABAergict transmission” and other neurotransmitters transmembrane transport and synaptic transmission of biological process. The subcellular localization of this study found that Chrm2 and Chrnb2 are mainly expressed in cell membranes as well as the peripheral secretory proteins of the membrane, providing a structural basis for enhancing the transport of substances in cells and the transmission of intercellular neurotransmitters. This study observed that knockdown of α-Syn after SCI promoted the recovery of neurological function in rats, possibly due to the reprogramming of the association between the nerves at the injury site by Chrm2 and Chrnb2, confirms their expression and cell localization, and may further clarify the bile after SCI in the future. In addition, Chrm2 and Chrnb2 subcellular localization are membrane proteins, which are more likely to become drugs and therapeutic targets, providing a structural basis for future clinical translation [44]. However, the role of the cholinergic system and its related immunoregulation is too broad, and the mechanism governing this phenomenon is complex. This study only initially found the relationship between the α-Syn, SCI and cholinergic pathways, and the specific in-depth mechanism warrants further study.

## Conclusions

Knockdown of α-synuclein after spinal cord injury enhance motor function and promote neurogenesis probably through enhancing cholinergic signaling pathways and neuroreceptor interactions. This study not only further clarifies the understanding of the mechanism of knockdown of α-synuclein on SCI but also helps to guide the treatment strategy for SCI.

## Methods

### Animals

A total of 45 SPF adult male Sprague-Dawley (SD) rats (Department of Animal Science, Peking University School of Medicine, Beijing, China) were used in this study. All the used rats were provided by the Department of Animal Science, Peking University Medical College, Beijing China. All rats were housed in separate cages under a 12-h light-dark cycle at 23 ± 1 °C and 50% relative humidity, and food and water were obtained ad libitum. All rats were acclimatized to the environment for at least 1 week prior to the experiment and maintained as directed by the experimental animal care and use guidelines. The study was approved by the Animal Welfare Ethics Branch of the Peking University Bioethics Committee. A completed ARRIVE guidelines checklist is included in Additional file 17: Checklist S1. Animals were randomized into three groups of 15 (*n* = 15) each: the CON group (i.e., the sham operation group), (2) the SCI group, and (3) the SCI + LV-*Snca*-shRNA group (abbreviated as the LV_SCI group), and the experimental design is shown in Fig. 1a.

### T3 spinal contusion model and lentiviral vector transfection knockdown of α-Syn

Lentiviral vector particles containing *Snca*-shRNA (NM_019169.2) were constructed and synthesized by Shan Dong ViGene Co., Ltd. (Shandong, China). The primers for *Snca* are as follows: forward: 5′-GTGGCTGCTGCTGAGAAAAC-3′, reverse: 5′ -TCCATGAACGACTCCCTCCT − 3′. The viral titer of LV-GFP-*Snca*-shRNA was 1.0 x 10E^9^ TU/ml. All rats received prophylactic antibiotic ampicillin sodium (80 mg/kg; Harbin Pharmaceutical Group Co., Ltd., China) for 3 days before SCI surgery. Rats were intraperitoneally injected with 2% sodium pentobarbital (0.1 ml/kg), and the C8-T4 dorsal skin was dissected along the T2 spine in vitro. The muscle tissue of the back was peeled off layer by layer. The thoracic T3 segment was dissected, and the lamina of the T3 segment was removed under a surgical microscope to expose the spinal cord. In the CON group, the incision was closed layer by layer only after the spinal cord was exposed, and the SCI group was struck with the PSI-IH precision striking device (IH impactor; Precision Systems and Instrumentation, Lexington, KY, USA) after the spinal cord was exposed. The strike strength was set to 400 Kdynes, the compression time was 5 s, and only one hit was employed. Following SCI, 10 μl of the target gene shRNA lentiviral vector was injected in situ (OI) at a dose of 10 μl with a titer of 1.0 x 10E9 TU/ml using a microsyringe. The CON group and the SCI group were given the same dose of blank lentiviral vector. Two days after surgery, rats were injected subcutaneously with Ringer’s sodium lactate solution (5 mL) and ampicillin sodium until the third day of injury. The rat bladder was squeezed daily 3 times after surgery until spontaneous urination was restored. All assessments and analyses were performed by researchers who were unaware of the experimental design but were experienced [45].

### Behavioral experiment

Basso, Beatlie, Bresnahan (BBB) motor function scores were used for the evaluation of hindlimb motor function [46]. The rats were placed on a circular platform with a diameter of 2 m. The walking and limb activity scores of the hind limbs were recorded. Each group was scored 1 day before surgery and on days 1, 7, 14, 21 and 28 after surgery. Rat body weight was measured daily.

### Tissue preparation

On the 28th day after surgery, all the rats were sacrificed, and a part of the spinal cord 5 mm above and below the T3 injury site was quickly placed in the cryotube for RNA-seq, and another part was verified by Western blotting and RT-qPCR. In addition, some tissues were used to make frozen slices. Timeline and grouping situation see Additional file 18: Figure S4.

### RNA extraction

Total RNA was extracted from the damaged spinal cord tissue using TRIzol® Reagent according the manufacturer’s instructions (Invitrogen), and genomic DNA was removed using DNase I (TaKara). Then, RNA quality was determined by 2100 Bioanalyzer (Agilent) and quantified using the ND-2000 (NanoDrop Technologies). Only high-quality RNA samples (OD260 / 280 = 1.8 ~ 2.2, OD260 / 230 ≥ 2.0, RIN ≥ 6.5, 28S:18S ≥ 1.0, > 10 μg) were used to construct a sequencing library.

### Library preparation and Illumina Hiseq xten sequencing

An RNA-seq transcriptome library was prepared following the TruSeq™ RNA sample preparation Kit from Illumina (San Diego, CA) using 5 μg of total RNA. Briefly, messenger RNA was isolated according to the polyA selection method by oligo (dT) beads and then fragmented by fragmentation buffer. Second, double-stranded cDNA was synthesized using a SuperScript double-stranded cDNA synthesis kit (Invitrogen, CA) with random hexamer primers (Illumina). Then, the synthesized cDNA was subjected to end-repair, phosphorylation and ‘A’ base addition according to Illumina’s library construction protocol. Libraries were size-selected for cDNA target fragments of 200–300 bp on 2% Low Range Ultra Agarose followed by PCR amplified using Phusion DNA polymerase (NEB) for 15 PCR cycles. After quantification by TBS380, a paired-end RNA-seq sequencing library was sequenced with the Illumina HiSeq xten (2 × 150-bp read length, Illumina, San Diego, CA, USA) supplied by Majorbio Biopharm Technology Co. Ltd. (Shanghai, China).

### Quality control, expression level and bioinformatics analysis

The raw paired end reads were trimmed and subjected to quality control by SeqPrep (https://github.com/jstjohn/SeqPrep) and Sickle (https://github.com/najoshi/sickle) with default parameters. The paired-end clean reads were aligned to the Rattus_norvegicus reference genome (Rnor_6.0) using the default parameters of TopHat (http://tophat.cbcb.umd.edu/, version2.1.1) [47]. Reference genome and gene model annotation files were downloaded from genome website (http://www.ensembl.org/Rattus_norvegicus/Info/Index) directly. RSEM (http://deweylab.biostat.wisc. Edu/rsem/) [48] was used to quantify gene abundances. To identify DEGs (differentially expressed genes) between two different samples, the expression level of each transcript was calculated based on the number of reads of transcripts per million readings (TPM, per million transcript readings). The readings were then normalized using DEseq2 v. 1.24.0 (http://bioconductor.org/packages/stats/bioc/DESeq2/) [49] and the statistical significance of the differentially expressed genes was assessed. The following criteria are used here: Default parameters: Benjamini & Hochberg (BH) p-adjust< 0.05 &|log2FC| ≥ 1 after multiple comparisons. In the initial data exploration, we constructed principal component analysis (PCA) to calculate the coefficient of variation between groups directly in R `prcomp`.

The genes and transcripts are aligned with six databases (NR, Swiss-Prot, Pfam, EggNOG, GO, and KEGG) to obtain comprehensive annotation information for genes and transcripts. The DIAMOND software (https://github.com/bbuchfink/diamond) [50] was used to sequence the genes and transcripts with the NR (ftp://ftp.ncbi.nlm.nih.gov/blast/db/), Swiss-Prot (ftp://ftp.uniprot.org/pub/databases), and EggNOG databases (http://eggnogdb.embl.de/#/app/home) [51]; Sequence alignment using BLAST2GO and GO database; Alignment with the Pfam database using HMMER software (ftp://selab.janelia.org/pub/software/hmmer3/3.0/hmmer-3.0.tar.gz) [52]; The results of KEGG Orthology were obtained using KOBAS2.1. The GO enrichment analysis was performed on the gene/transcript of the gene set using the software Goatools (https://github.com/tanghaibao/Goatools) [53], using Fisher’s exact test. In order to control the calculation of false positives, Goatools was used to perform BH for multiple test to correct the *P* value. When the corrected *P* value (FDR) was < 0.05, it was considered that the GO function was significantly enriched. The KEGG PATHWAY enrichment analysis was performed on the genes in the gene set using the R script of KOBAS (http://kobas.cbi.pku.edu.cn/home.do) [54]. When the corrected *P* value (P) is < 0.05, it is considered that this KEGG PATHWAY function is significantly enriched.

### Real-time quantitative PCR (RT-qPCR) verification

Real-time Quantitative PCR, total RNA was first extracted from spinal cord injury tissue of TRIzol (Invitrogen, Thermo Fisher Scientific Inc., USA) according to the manufacturer’s protocol. The ratio of absorbance of A260/280 and A260/230 was then determined using an ultraviolet-visible spectrophotometer (Nanodrop 2000, Thermo Fisher Scientific, Waltham, MA, USA) to determine the purity and concentration of total RNA for each sample. Total RNA (2 μg/sample) was reacted with a fastking cDNA first strand synthesis kit (Tiangen, Beijing, China) to synthesize cDNA, and then real-time reaction was carried out using SYBR Green PCR Master Mix (Tiangen, Beijing, China). The expression level of glyceraldehyde 3-phosphate dehydrogenase (GAPDH) was used as an internal control. Three replicate wells were set up for all reaction samples and repeated three times. All primers used in this experiment were supplied by Sangon Biotech (Shanghai) Co., Ltd. (Additional file 16: Table S13 listed the primer sequences). Amplification was performed by QuantStudio Design and Analysis software (Applied Biosystems), and the melting curve analysis confirmed primer specificity. Finally, the cycle threshold (CT) fluorescence value was determined. The data were analyzed by a -ΔΔCT method by researchers blinded to the grouping of each animal.

### Western blotting

Fresh spinal cord injured tissue was homogenized in RIPA buffer (including protease inhibitor cocktail). Add 20 μg of total protein and separate in a 15% SDS-PAGE gel, then transfer to polyvinylidene fluoride (PVDF). Blocking with 5% skim milk in Tris buffered saline (150 mM NaCl, 0.1% Tween 20, 20 mM Tris, pH 7.4), rabbit anti-Alpha-synuclein antibody (1:1000, GTX112799, GeneTex, Inc., USA) was used overnight at 4 °C. The horseradish peroxidase-conjugated secondary antibody solution (1:10000, Boster Biological Technology, Ltd. wuhan China) was then treated for 1 h, washed, and placed in 1 ml ECL substrate (Millipore, Billerica MA, USA). Quantitative image analysis was performed with ImageJ software (NIH, Bethesda, MD, USA). Results are expressed as relative densities.

### Bromodeoxyuridine (BrdU) administration

To label proliferating cells after spinal cord injury, rats were injected intraperitoneally (100 mg / kg) with the thymidine analogue bromodeoxyuridine (BrdU; 10 mg / mL in sterile saline; Sigma-Aldrich, 7 days prior to sampling) St. Louis, MO, USA). Each group of rats was given a specific BrdU pulse protocol [55].

### Immunofluorescence

Frozen sections were thawed for 30 min at room temperature and washed three times in 0.1 mmol / L PBS (PBS-TX) containing 0.1% Triton X-100 (Sigma-Aldrich, St. Louis, MO, USA) (10 min each) and preincubated for 15 min at 37 °C in permeabilization blocking buffer (0.1 mmol / L PBS, pH 7.3, 0.5% Triton). The sections were blocked with 10% (v/v) goat serum (Boster Biological Technology, Ltd., Wuhan, China) for another 30 min. The sections were then incubated overnight with the primary antibody at 4 °C. After washing with PBS-TX for the next day (3 times, 10 min each), the second antibody was incubated for 1 h at room temperature and then washed with PBS-TX. The nuclei were stained with 4,6-diamidino-2-phenylindole (DAPI, 1 μg/mL; Sigma-Aldrich) for 5 min, washed with PBS-TX and sealed with an anti-fluorescence quencher. Images were captured under confocal fluorescence microscopy and a Leica DM4 B fluorescence microscope (Leica Microsystems Inc., Wetzlar, Germany) by Leica TCS SP8 (Leica Microsystems Inc., Wetzlar, Germany). Negative controls were performed with the corresponding isotype serum instead of the primary antibody. Dilution was performed using the following antibodies and antibody dilutions: primary antibodies included mouse anti-DCX (1:100; Abcam, Cambridge, MA), rabbit anti-Chrm2 (1:100; Biosynthesis Biotechnology Inc. Beijing, China), mouse anti-BrdU (1:100; Abcam, Cambridge, MA), and rabbit anti-Chrnb2 (1:400; Abcam, Cambridge, MA), rabbit anti-Alpha-synuclein (1:1000, GeneTex, Inc., USA). Fluorescent secondary antibodies included Alexa Fluor 647-conjugated AffiniPure goat anti-rabbit IgG (H + L) (1:800; Jackson ImmunoResearch Laboratories, West Grove, PA) and Cy3-conjugated goat anti-mouse secondary antibody (1:200; Boster Biological Technology, Ltd., Wuhan, China).

### Statistical analysis

Data are expressed as the mean ± S.D. Statistical analysis was assessed by GraphPad Prism 7.0 (GraphPad Software Inc., San Diego, CA). Comparisons between the two groups were performed using Student’s t-test or the Mann-Whitney test as appropriate. One-way and two-way ANOVA and multiple comparisons of Tukey were used between groups. The BBB motor function score was analyzed by two-way repeated measurements of analysis of variance (ANOVA) followed by Holm-Sidak multiple comparison test. A *P*-value < 0.05 was considered statistically significant. * *p* < 0.05, ** *p* < 0.01, *** *p* < 0.001, and **** *p* < 0.0001.

## Supplementary information

**Additional file 1: Table S1.** Summary of Illumina’s postsequencing sequence assembly.

**Additional file 2: Table S2.** Mapping results between transcriptome and reference genome.

**Additional file 3: Table S3.** List of 210 upregulated DEGs expressed between the SCI group and the LV_SCI group.

**Additional file 4: Table S4.** List of 127 downregulated DEGs expressed between the SCI group and the LV_SCI group.

**Additional file 5: Table S5.** The significant of GO terms enriched between the SCI group and the LV_SCI group 210 upregualted DEGs.

**Additional file 6: Table S6.** The significant of GO terms enriched between the SCI group and the LV_SCI group 127 downregualted DEGs.

**Additional file 7: Table S7.** The significant GO terms of exclusive 153 DEGs in LV_SCI group compared to the other two groups.

**Additional file 8: Figure S1.** The heat map for cluster analysis and The heat map for cluster analysis

**Additional file 9: Table S8.** KEGG pathway enriched between the SCI and the LV_SCI 210 upregulated DEGs.

**Additional file 10: Table S9.** The detail information of 21 differential genes derived from Fig. 4-B and Fig. 6a.

**Additional file 11: Figure S2.** KEGG mapping of neuroactive ligand-receptor interaction

**Additional file 12: Table S10.** KEGG pathway enriched bewteen the SCI and the LV_SCI 127 downregulated genes.

**Additional file 13: Table S11.** KEGG pathway enriched the differentially expressed genes (CON VS SCI upregulated genes).

**Additional file 14: Table S12.** KEGG pathway enriched the differentially expressed genes (CON VS SCI downregulated genes).

**Additional file 15: Figure S3.** The KEGG Enrichment Analysis (CON vs SCI).

**Additional file 16: Table S13.** List of 22 genes primers used for RNA-Seq data validation.

**Additional file 17: Checklist S1.** Completed “The ARRIVE Guidelines Checklist” for reporting animal data in this manuscript.

**Additional file 18: Figure S4.** Timeline and grouping situation of experiment rats.

## Data Availability

RNA-Seq raw data have been deposited in the NCBI Sequence Read Archive (SRA, https://submit.ncbi.nlm.nih.gov/subs/sra/SUB5917896/overview). Accession IDs for *Rattus norvegicus* BioProject = PRJNA552942;BioSample = SAMN12222084 - SAMN12222092 (9 objects); SRA = SRR9448449 - SRR9448455(9 objects)

## Abbreviations

BBB: Basso, Beatlie, Bresnahan
BrdU: Bromodeoxyuridine
Chrm2: Muscarinic M2 Cholinergic Receptor
Chrnb2: Cholinergic Receptor Nicotinic Beta 2 Subunit
CNS: Central Nervous System
LV: Lentivirus
mAChR: Muscarinic Acetylcholine Receptor
nAChR: Nicotinic Acetylcholine Receptor
RNA-seq: RNA Sequencing
RT-qPCR: Real-time Quantitative Polymerase Chain Reaction
SCI: Spinal Cord Injury
shRNA: Short hairpin RNA
VNS: Vagus Nerve Stimulation
α-Syn: α-Synuclein
