# Supplementary material for: Transcriptomic analysis of α-synuclein knockdown after T3 spinal cord injury in rats
Source: BMC Genomics. 2019 Nov 14;20:851. doi: 10.1186/s12864-019-6244-6 (PMC6854783; doi:10.1186/s12864-019-6244-6)
Supplement: Supplementary file 8 — Additional file 8: Figure S1. The heat map for cluster analysis and The heat map for cluster analysis [file 12864_2019_6244_MOESM8_ESM.pdf]

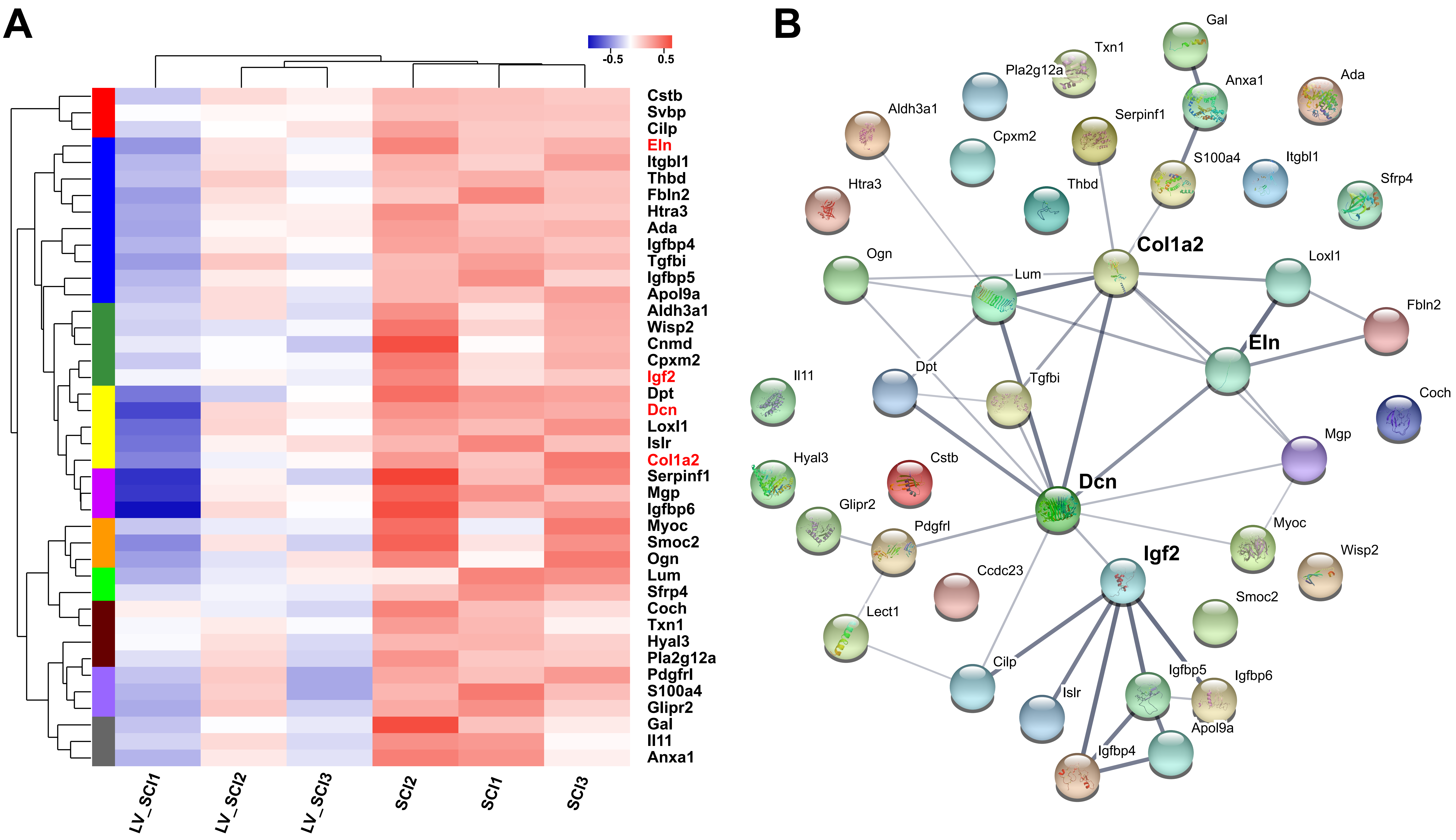

Additional file 8: Figure S1.

(A) The heat map for cluster analysis

(B) Protein protein interaction network diagram
