# Supplementary material for: Transcriptomic analysis of α-synuclein knockdown after T3 spinal cord injury in rats
Source: BMC Genomics. 2019 Nov 14;20:851. doi: 10.1186/s12864-019-6244-6 (PMC6854783; doi:10.1186/s12864-019-6244-6)
Supplement: Supplementary file 11 — Additional file 11: Figure S2. KEGG mapping of neuroactive ligand-receptor interaction [file 12864_2019_6244_MOESM11_ESM.pdf]

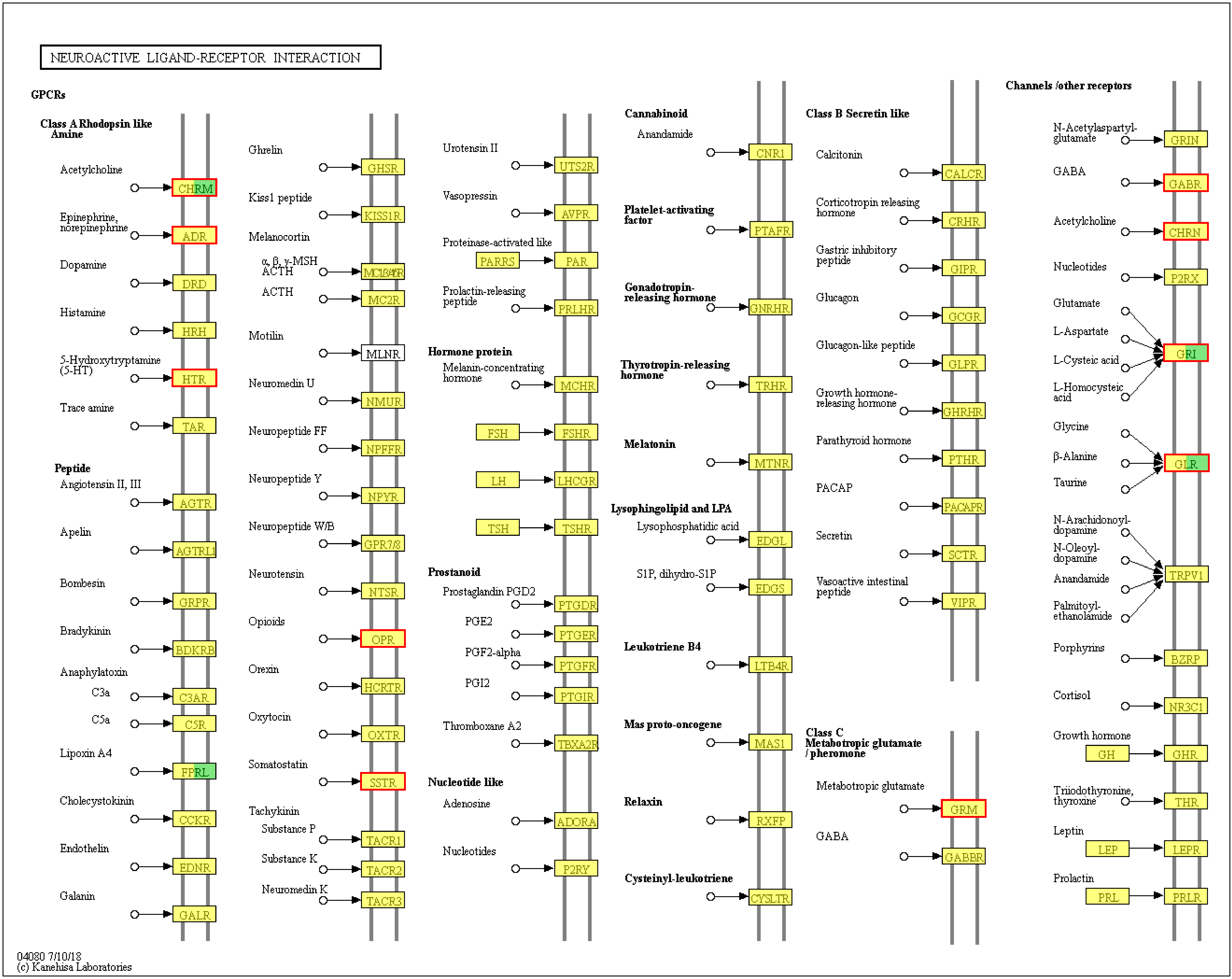

Additional file 11: Figure S2.

Figure S2. KEGG mapping of neuroactive ligand-receptor interaction

Note: All products with a color background in the figure belong to the KEGG background annotation results of the project gene/transcript.
The yellow background indicates the reference gene/transcript, the green background indicates the new gene/transcript,
and the yellow and green background indicates both the existing reference gene/transcript and the new gene/transcript.
The red border indicates that the gene/transcript of the gene set is annotated.
