# Supplementary figures and images for: Transcriptomic analysis of α-synuclein knockdown after T3 spinal cord injury in rats
Source: BMC Genomics. 2019 Nov 14;20:851. doi: 10.1186/s12864-019-6244-6 (PMC6854783; doi:10.1186/s12864-019-6244-6)

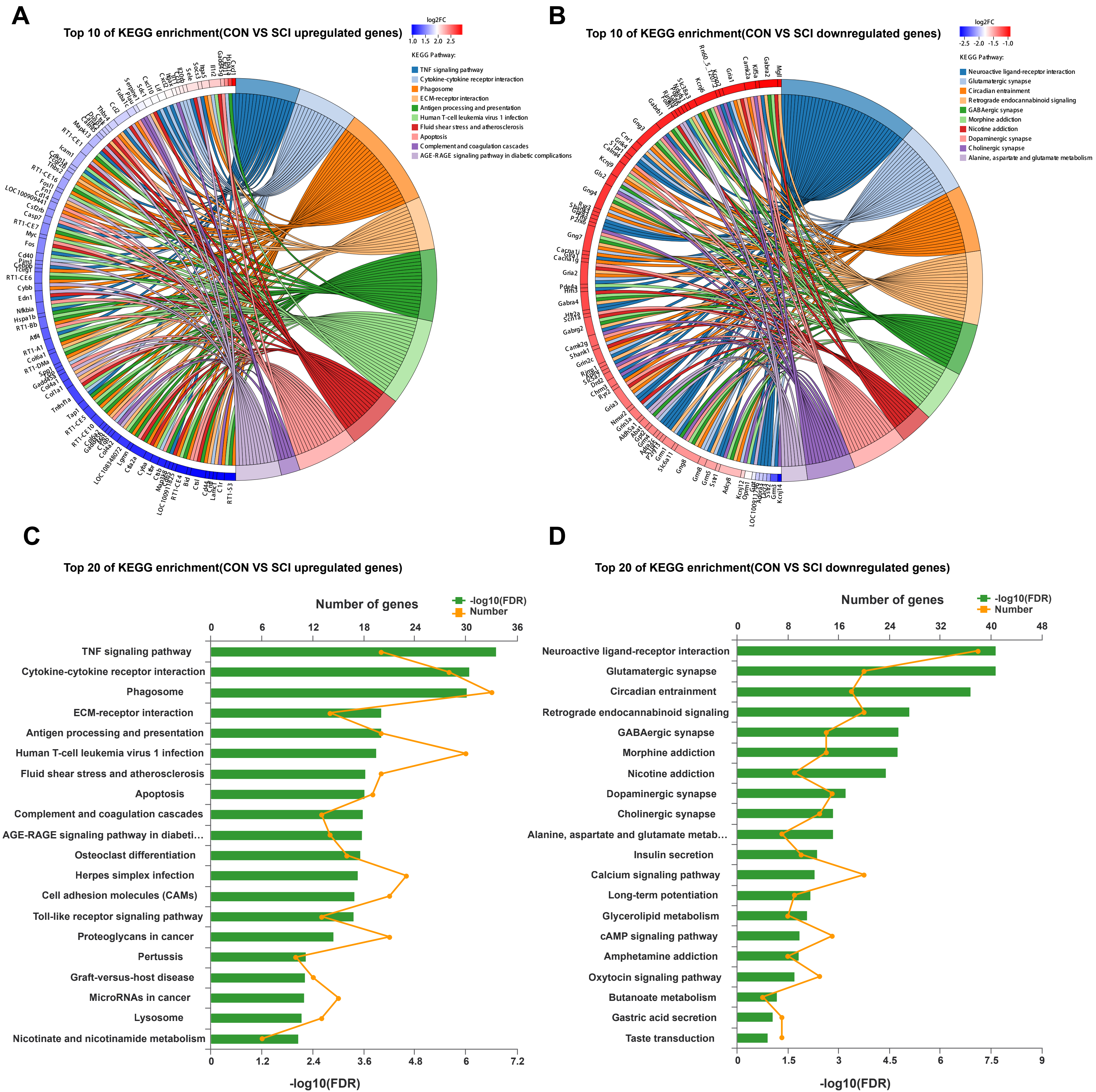

Additional File 15: Figure S3 The KEGG Enrichment Analysis (CON VS SCI)

Supplement: Supplementary file 15 — Additional file 15: Figure S3. The KEGG Enrichment Analysis (CON vs SCI). [file 12864_2019_6244_MOESM15_ESM.pdf]

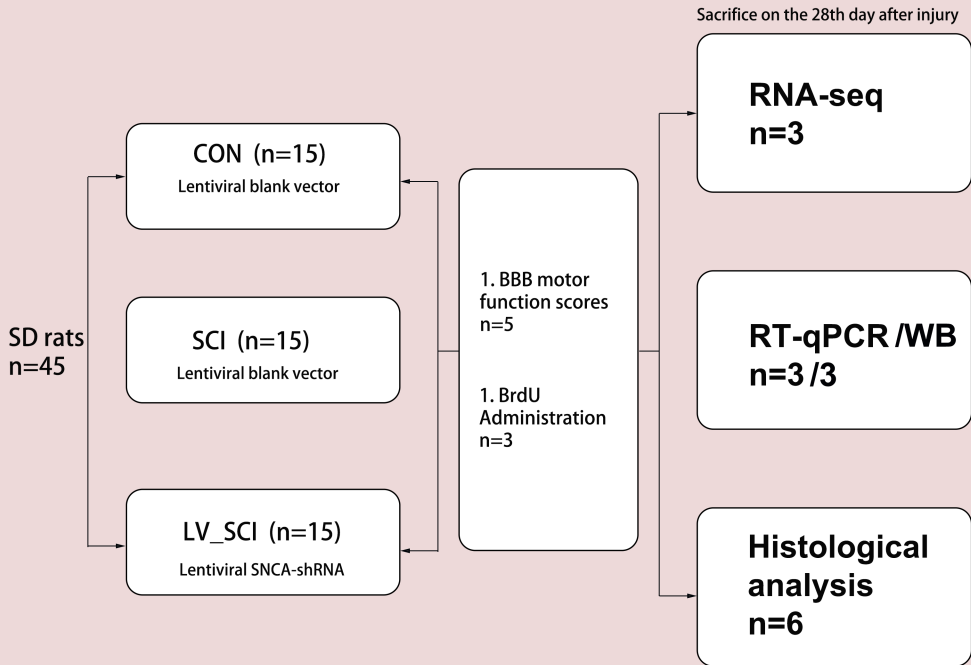

**Additional file 18: Figure S4** Timeline and grouping situation of experiment rats.

Supplement: Supplementary file 18 — Additional file 18: Figure S4. Timeline and grouping situation of experiment rats. [file 12864_2019_6244_MOESM18_ESM.pdf]
