# Supplementary material for: Transcriptomic analysis of α-synuclein knockdown after T3 spinal cord injury in rats
Source: BMC Genomics. 2019 Nov 14;20:851. doi: 10.1186/s12864-019-6244-6 (PMC6854783; doi:10.1186/s12864-019-6244-6)
Supplement: Supplementary file 16 — Additional file 16: Table S13. List of 22 genes primers used for RNA-Seq data validation. [file 12864_2019_6244_MOESM16_ESM.pdf]

Table S 13. List of 22 genes primers used for RNA-Seq data validation.

NO.	Rat gene	Forward primer (5′ -3′)	Reverse primer (5′ -3′)
1	Glra1	5' GTAAGTGCCCATCGGAAAC 3'	5' CCAAAGGTAGAATCGCAG 3'
2	Htr2a	5' TGGATTTACCTGGATGTGC 3'	5' TGTGGATGGACCGTTGGAAG 3'
3	Kcnj14	5' CGCTACCTAAGTGACCTGTT 3'	5' AGTGTCTCGTTGCGTTTC 3'
4	Slc18a3	5' GTGCGAGGACGACTACAACTAT 3'	5' GAAGGATTCCAGCAAAGG 3'
5	Chat	5' GCAAGACTGCGAGAACTAA 3'	5' TCACCGCTTCAAACAACT 3'
6	Chrnb2	5' TCATCATTCGTCGCAAAC 3'	5' TAGCAGCACAGAAATACAAAGT 3'
7	Chrm2	5' GGGCAAGCAAGAGTAGAAT 3'	5' GAGTGGTCCAGCGAAGTG 3'
8	Kcnj12	5' TCTTGCCTCCTGGTTGCT 3'	5' TGGGATGTACTCACCCTCTT 3'
9	Grm3	5' TGGGTTTGATACTGGTGC 3'	5' CTGAGAATAGGTGGTTGC 3'
10	Grik3	5' CACCGACCATCTCCATAGC 3'	5' CTTTCGTTCGCATTCCTG 3'
11	Gabrb2	5' TGCTAATGCCAACAACG 3'	5' CTCCTCAGGCGACTTTT 3'
12	Slc5a7	5' TAGATCTCACAGCGAAGCAGG 3'	5' TGGCACTGAGCATTTGACAG 3'
13	Adra1d	5' CGACATCCTGAGCGTCACTT 3'	5' CGTACCGGTCCACAGAGATG 3'
14	Gria3	5' AACCCTAAAGGCTCAGCAT 3'	5' TATAGAACACGCCTGCCACA 3'
15	Grm1	5' CACACTCGGACAAAATCTA 3'	5' CGATGACTTCATCTCTGTCT 3'
16	Grm4	5' AGTGACAACAGCCGCTATGAC 3'	5' CACACACCTCCGTTCTCTCG 3'
17	Sstr1	5' CTA CTT TGC CGC CTG GTG CTC 3'	5' TGG CAA TGA TGA GCA CGT AAC3'
18	Gabrg2	5' GAAAAACCCTGCCCCTACCA 3'	5' TGCGAATGTGTATCCTCCCG 3'
19	Chrna4	5' CCTGACTTGAAGACATCA 3'	5' CACAGTACTGGATACTCC 3'
20	Oprm1	5' TTCCTGGTCATGTATGTGATTGT 3'	5' GGGCAGTGTACTGGTCGCTAA 3'
21	Kcnq2	5' CGTTCATCTACCACGCCTAC 3'	5' TACTCCACGCCAAACACCAC3'
22	Gapdh	5'GGCACAGTCAAGGCTGAGAATG-3'	5'ATGGTGGTGAAGACGCCAGTA3'
